# Terminal S Wave Pattern in Lead V3 as a Simple Marker for Ventricular Tachycardia Risk Stratification in Patients With Repaired Tetralogy of Fallot

**DOI:** 10.1111/jce.70468

**Published:** 2026-08-10

**Authors:** Yuki Matsuoka, Miyako Igarashi, Naoto Kawamatsu, Yuto Iioka, Yoshihiro Harano, Yuki Komatsu, Tomoko Machino‐Ohtsuka, Hiro Yamasaki, Tomoko Ishizu

**Affiliations:** ^1^ Department of Cardiology, Institute of Medicine University of Tsukuba Tsukuba Japan

**Keywords:** anatomical isthmus, electro‐anatomical mapping, electrocardiogram, risk stratification, Tetralogy of Fallot, ventricular tachycardia

## Abstract

**Introduction:**

Patients with repaired tetralogy of Fallot (rTOF) remain at risk of ventricular tachycardia (VT). This risk is most commonly related to slow conduction within the septal isthmus, which is defined as the region between the ventricular septal defect (VSD) patch and adjacent valvular annuli. While assessment of septal isthmus conduction currently requires invasive electrophysiological mapping, the 12‐lead electrocardiogram (ECG) may offer a noninvasive alternative. Therefore, the present study aimed to identify ECG markers reflecting septal isthmus conduction properties and evaluate their ability to predict isthmus conduction characteristics and VT inducibility.

**Methods and Results:**

In five patients with rTOF undergoing septal isthmus ablation, 12‐lead ECGs obtained before and after ablation were compared to identify candidate ECG markers. The identified marker was validated in an independent cohort of 23 patients stratified according to septal isthmus conduction properties into normal conduction (Normal, *n* = 5), slow‐conducting isthmus (SCI, *n* = 10), and conduction block (Block, *n* = 8) groups. The results indicated that the terminal S wave (TSW) in lead V3 became more prominent after ablation compared with that before ablation. In the validation cohort, V3 TSW was categorized as absent, small (< 0.1 mV), or large (≥ 0.1 mV), and the TSW patterns differed significantly among the groups (*p* < 0.001). Specifically, absent TSW was dominant in the Normal group (4/5, 80%), small TSW was the most frequent in the SCI group (7/10, 70%), and large TSW was predominant in the Block group (7/8, 87.5%). Small TSW predicted SCI with 70% sensitivity and 85% specificity (*p* = 0.013), whereas large TSW predicted Block with 88% sensitivity and 80% specificity (*p* = 0.006). Moreover, large TSW predicted VT non‐inducibility with high specificity and high positive predictive value (*p* = 0.019).

**Conclusion:**

This study indicates that V3 TSW is a simple, noninvasive ECG marker reflecting septal isthmus conduction properties in rTOF, suggesting its potential utility for VT risk stratification.

## Graphical abstract

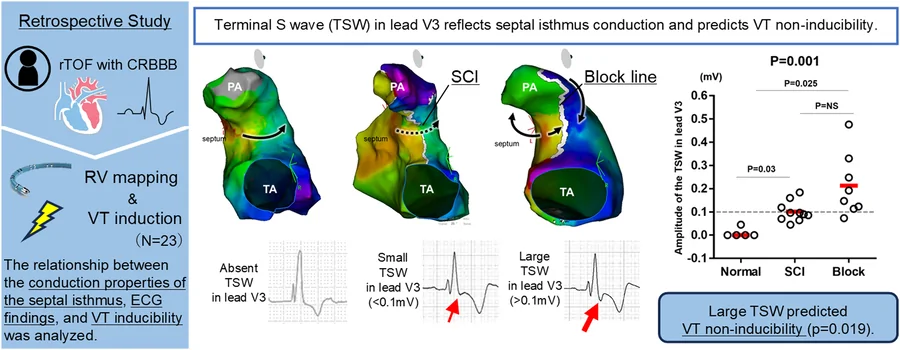

## 1 Introduction

In patients with repaired tetralogy of Fallot (rTOF), ventricular tachycardia (VT) is commonly related to slow conduction through surgically created isthmuses [1, 2, 3, 4, 5, 6, 7, 8, 9, 10, 11]. Anatomical isthmuses are classified based on their location relative to surgical scars, patches, and valvular annuli: (1) between the tricuspid annulus and the right ventricular (RV) outflow tract patch or RV incision; (2) between a non‐transannular incision and the pulmonary annulus; (3) between the ventricular septal defect (VSD) patch and the pulmonary annulus; and (4) between the VSD patch and the tricuspid annulus [8, 12]. Among these, septal isthmuses involving the VSD patch have emerged as the predominant substrates for VT in the contemporary surgical era [8, 12]. Slow‐conducting or blocked isthmuses can be identified using invasive electro‐anatomical mapping; however, this approach is impractical for routine risk assessment. Moreover, the increasing use of transcatheter pulmonary valve implantation (TPVI) has further heightened the need for pre‐procedural RV substrate evaluation, as valve implantation may limit subsequent catheter access to septal isthmuses.

At our institution, RV mapping and VT induction are performed before TPVI for risk assessment, with ablation limited to patients demonstrating inducible sustained monomorphic VT. In the present study, we hypothesized that linear ablation across the septal isthmus that results in conduction block could produce distinctive surface electrocardiogram (ECG) changes reflecting alterations in RV conduction. Distinguishing slow‐conducting from blocked septal isthmuses is of particular clinical relevance, because slow‐conducting isthmuses may serve as critical components of VT circuits, whereas blocked isthmuses are less likely to participate in reentrant VT circuits. Therefore, we aimed to identify ECG markers of septal isthmus conduction properties to provide a simple, noninvasive method for assessing arrhythmic substrate and stratifying VT risk in patients with rTOF.

## 2 Methods

### 2.1 Study Population and Study Design

Patients with rTOF undergoing evaluation for TPVI or surgical pulmonary valve replacement (PVR) at our institution between January 2023 and April 2026 were analyzed. Only patients with complete right bundle branch block (CRBBB) were included, whereas those with narrow QRS or permanent ventricular pacing were excluded. RV substrate mapping and programmed VT induction were performed as part of the preoperative risk assessment before PVR/TPVI in all patients. Patients with inducible sustained monomorphic VT subsequently underwent catheter ablation and were also included in the study.

The study was conducted in two steps: an exploratory retrospective analysis and a validation cohort study. In the exploratory retrospective analysis, 12‐lead ECGs obtained before and after ablation in the first five patients with ablation‐proven conduction block across the septal isthmus were compared to identify ECG markers reflecting isthmus conduction [8, 12, 13]. These five exploratory cases were excluded from the validation cohort analysis, in which the identified marker was evaluated in an independent cohort of 23 patients to assess its association with septal isthmus conduction and VT inducibility.

This study complied with the Declaration of Helsinki and was approved by the University of Tsukuba Ethics Committee (R07‐027). The requirement for written informed consent was waived by the institutional review board, and an opt‐out method was used.

### 2.2 RV Mapping and VT Induction

Electro‐anatomical mapping was performed using the CARTO system with a DecaNav catheter (Biosense Webster, Diamond Bar, CA, USA) during sinus rhythm. Isthmus length was measured as the distance between normal bipolar electrograms on both sides (voltage > 1.5 mV), and conduction time was measured as the difference in local activation time. Conduction velocity was calculated as the length of isthmus divided by the conduction time [8, 14]. A velocity of 0.5 m/s or greater was defined as normal conduction, less than 0.5 m/s as a slow conduction isthmus (SCI), and absence of conduction as a block. Programmed stimulation with up to triple extrastimuli was performed from the RV apex and outflow tract at a basic cycle length of 400 ms, with a minimum coupling interval of 180 ms. All procedures were performed under conscious sedation.

### 2.3 Catheter Ablation

In patients in whom sustained monomorphic VT was induced, catheter ablation was performed before TPVI using a 3.5‐mm tip, contact force–sensing, open‐irrigated catheter (QDOT MICRO; Biosense Webster). Linear lesions were created along the endocardial RV to achieve bidirectional conduction block across the septal isthmus. Additional ablation from the contralateral coronary cusps was delivered if the block was incomplete or VT remained inducible. After ablation, stimulation confirmed non‐inducibility of VT, and remapping verified conduction block.

### 2.4 Electrocardiogram Analysis

Standard 12‐lead ECGs were recorded at 25 mm/s, 10 mm/mV using either the Bard LABSYSTEM PRO EP Recording System (Boston Scientific, Marlborough, MA, USA) or the Prime Vita system (Nihon Kohden Inc., Tokyo, Japan) during the ablation procedure and with ECG‐2550 (Nihon Kohden Inc.) in the validation study. The standard 12‐lead ECG obtained before electro‐anatomic mapping and VT induction was used for the validation cohort analysis. Recordings were analyzed by two independent physicians blinded to the mapping results.

### 2.5 Electrocardiogram Parameters

A notched R in lead V1 and the presence of a terminal S wave (TSW) in lead aVF were assessed using established definitions [14, 15]. The TSW in the precordial leads was defined as a terminal negative deflection of the QRS. Its amplitude was measured from the nadir to the ST junction and categorized as absent, small (< 0.1 mV), or large (≥ 0.1 mV) (Figure S1).

### 2.6 Statistical Analysis

Continuous variables are expressed as mean ± standard deviation or median with interquartile range, as appropriate. Categorical variables are expressed as numbers and percentages. Comparisons between two groups were performed using the Student's *t*‐test or Mann–Whitney *U* test, as appropriate. Comparisons among three groups were performed using one‐way analysis of variance (ANOVA) with Tukey's post‐hoc test or the Kruskal–Wallis test with Dunn–Bonferroni post‐hoc correction, as appropriate. Categorical variables were compared using the *χ* ^2^ test or Fisher's exact test, as appropriate. Receiver operating characteristic (ROC) curve analysis was used to determine the optimal TSW cutoff for predicting septal isthmus conduction, and the area under the curve (AUC), sensitivity, and specificity were calculated. A two‐sided *p*‐value < 0.05 was considered statistically significant. Statistical analyses were performed using SPSS (version 31; IBM SPSS Statistics, Armonk, NY, USA).

## 3 Results

### 3.1 Study Population

A total of 28 patients with rTOF underwent right ventricular mapping and VT induction testing. Sustained monomorphic VT was inducible in 11 patients. The exploratory retrospective analysis included the first five patients who underwent septal isthmus ablation, whose ECG findings before and after ablation were compared to identify potential ECG markers of septal isthmus conduction. The remaining 23 patients were enrolled in the independent validation cohort study. Baseline characteristics of the validation cohort are summarized in Table 1.

**Table 1:** Baseline characteristics of patients in the validation cohort.

	Conduction pattern in septal isthmus			*p* value
	Normal (*n* = 5)	SCI (*n* = 10)	Block (*n* = 8)	
Age, years	44.8 ± 6.1	39.8 ± 15.6	38.6 ± 13.4	NS
Sex, male (%)	3 (60)	5 (50)	4 (50)	NS
History of sustained VT/VF (%)	1 (20)	1 (10)	0 (0)	NS
History of AT/AF (%)	3 (60)	2 (20)	5 (62.5)	NS
Corrective surgery findings				
Age at surgery, years	3.4 ± 2.7	4.4 ± 3.5	6.6 ± 5.3	NS
Time since surgery, median (IQR), years	43 [39–43]	38 [26–47]	32 [29–33]	NS
Type of surgery				
Transannular patch (%)	3 (60)	6 (60)	7 (87.5)	NS
non‐transannular patch (%)	2 (40)	4 (40)	1 (12.5)	
Conduit (%)	0 (0)	0 (0)	0 (0)	
ECG findings				
AT/AF (%)	0 (0)	0 (0)	0 (0)	NS
QRS duration, ms	145.0 ± 19.6	147.9 ± 11.7	163.5 ± 27.9	NS
fragmented QRS (%)	1 (20)	5 (50)	5 (62.5)	NS
Echocardiography findings				
LVEF, %	57.8 ± 6.9	58.1 ± 4.0	55.5 ± 7.9	NS
RVEDA, cm^2^	41.2 ± 10.7	33.5 ± 8.5	38.6 ± 11.1	NS
FAC, %	35.6 ± 7.7	40.5 ± 8.8	38.0 ± 9.7	NS
PR (moderate ~ severe) (%)	5 (100)	5 (50)	8 (100)	0.012
VT induction (%)	2 (40)	4 (40)	0 (0)	NS

Abbreviations: AF, atrial fibrillation; AI, anatomical isthmus; AT, atrial tachycardia; FAC, fractional area change; IQR, Interquartile Range; LVEF, left ventricular ejection fraction; PR, pulmonary regurgitation; RV, right ventricle; RVEDA, right ventricular end diastolic area; SCI, slow conducting isthmus; VF, ventricular fibrillation; VT, ventricular tachycardia.

### 3.2 Exploratory Retrospective Analysis

All induced VTs in the five exploratory cases were confirmed or suggested to be septal anatomical isthmus‐related by 3D electro‐anatomical mapping. We compared the 12‐lead ECGs obtained before and after the procedure among the five patients (Supporting Information S1: Table 1). We systematically compared previously reported parameters, including S wave fragmentation in lead I, the TSW in lead aVF, and the presence of a notched R in lead V1, as well as other ECG changes, between before and after ablation data.

Before ablation, large TSWs were common in lead V4, whereas they were infrequent in lead V3. After ablation, large TSWs became common in the precordial leads, with consistent involvement of leads V3 and V4 in all patients. Following confirmation of complete conduction block across the septal isthmus, a TSW consistently emerged and deepened in lead V3 in all five exploratory cases. A TSW amplitude of ≥ 0.1 mV was observed in all patients (Figures S2 and S3). These findings suggest that pronounced TSW in lead V3 reflects the conduction properties of the septal isthmus.

### 3.3 Validation Study

Among the 23 patients included in the validation cohort, the presence of septal anatomical isthmuses related to the VSD patch was assessed using available surgical records, CT findings, and 3D electro‐anatomical mapping. Anatomical isthmus 3 was present in all patients (23/23, 100%), whereas anatomical isthmus 4 was present in four patients (17.4%). The conduction property of the septal isthmus (comprising anatomical isthmuses 3 and 4) was classified as normal conduction (Normal, *n* = 5), slow‐conducting isthmus (SCI, *n* = 10), or complete conduction block (Block, *n* = 8) (Figure 1). Detailed data on the prevalence and conduction properties of anatomical isthmuses in both the exploratory and validation cohorts are provided in Supporting Information S1: Table 2.

**Figure 1:**
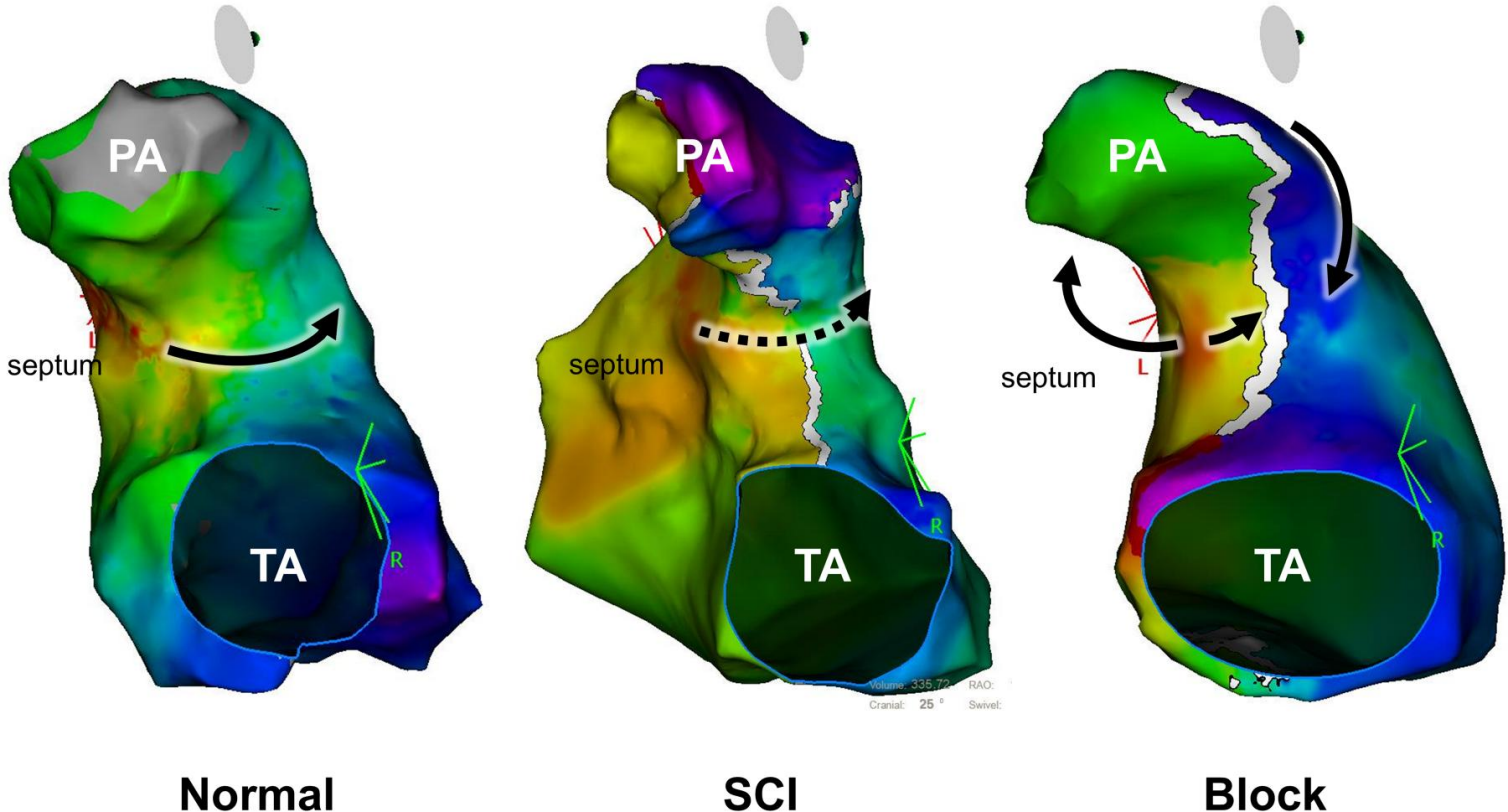
Representative right ventricular activation maps demonstrating conduction properties of the septal isthmus. Left panel: Normal septal isthmus conduction with uniform propagation (conduction velocity 0.7 m/s, black arrow). Middle panel: Slow‐conducting isthmus (SCI). The solid white line indicates regions with activation time differences, suggestive of conduction block. The gap reveals slow conduction, with a conduction velocity of 0.17 m/s (black dotted arrow). Right panel: Complete block of the septal isthmus. The septal wavefront is blocked by the white line on the posterior wall, propagates around the anterior wall, and returns along the free wall (black arrow). PA, pulmonary annulus; SCI, slow‐conducting isthmus; TA, tricuspid annulus.

We categorized TSW in lead V3 as absent, small (< 0.1 mV), or large (≥ 0.1 mV). TSW was absent in 80% of the Normal group, small in 70% of the SCI group, and large in 87.5% of the Block group (*p* < 0.001; Table 2). Median TSW amplitude increased progressively across the three groups (0, 0.09, and 0.17 mV, respectively; *p* = 0.001). Pairwise comparisons with Dunn–Bonferroni correction showed that TSW amplitude was significantly greater in the SCI (*p* = 0.031) and Block groups (*p* = 0.025) than in the Normal group, whereas the difference between the SCI and Block groups was not statistically significant (*p* = 0.173) (Figure 2A). ROC curve analysis revealed that the TSW amplitude in lead V3 predicted conduction block with an AUC of 0.88 (95% confidence interval [CI] 0.74–1.00, *p* < 0.001). The optimal cutoff was 0.11 mV (sensitivity 88%, specificity 80%), supporting the provisional cutoff of 0.1 mV established in the exploratory study is acceptable as a practical threshold. Using this threshold, a small TSW (< 0.1 mV, excluding 0 mV) predicted SCI with a sensitivity of 70% and a specificity of 85%, whereas a large TSW (≥ 0.1 mV) predicted conduction block with a sensitivity of 88% and a specificity of 80% (Table 3). Additional thresholds (e.g., 0.2 mV) and their diagnostic performance are summarized in Table 3.

**Table 2:** Relationship between the septal isthmus conduction property and TSW in lead V3.

		TSW pattern in lead V3		
		Absent	Small (< 0.1 mV)	Large (≥ 0.1 mV)
Normal	*n* = 5	4 (80%)	1 (20%)	0 (0%)
SCI	*n* = 10	0 (0%)	7 (70%)	3 (30%)
Block	*n* = 8	0 (0%)	1 (12.5%)	7 (87.5%)

Abbreviations: SCI, slow‐conducting isthmus; TSW, terminal S wave.

**Figure 2:**
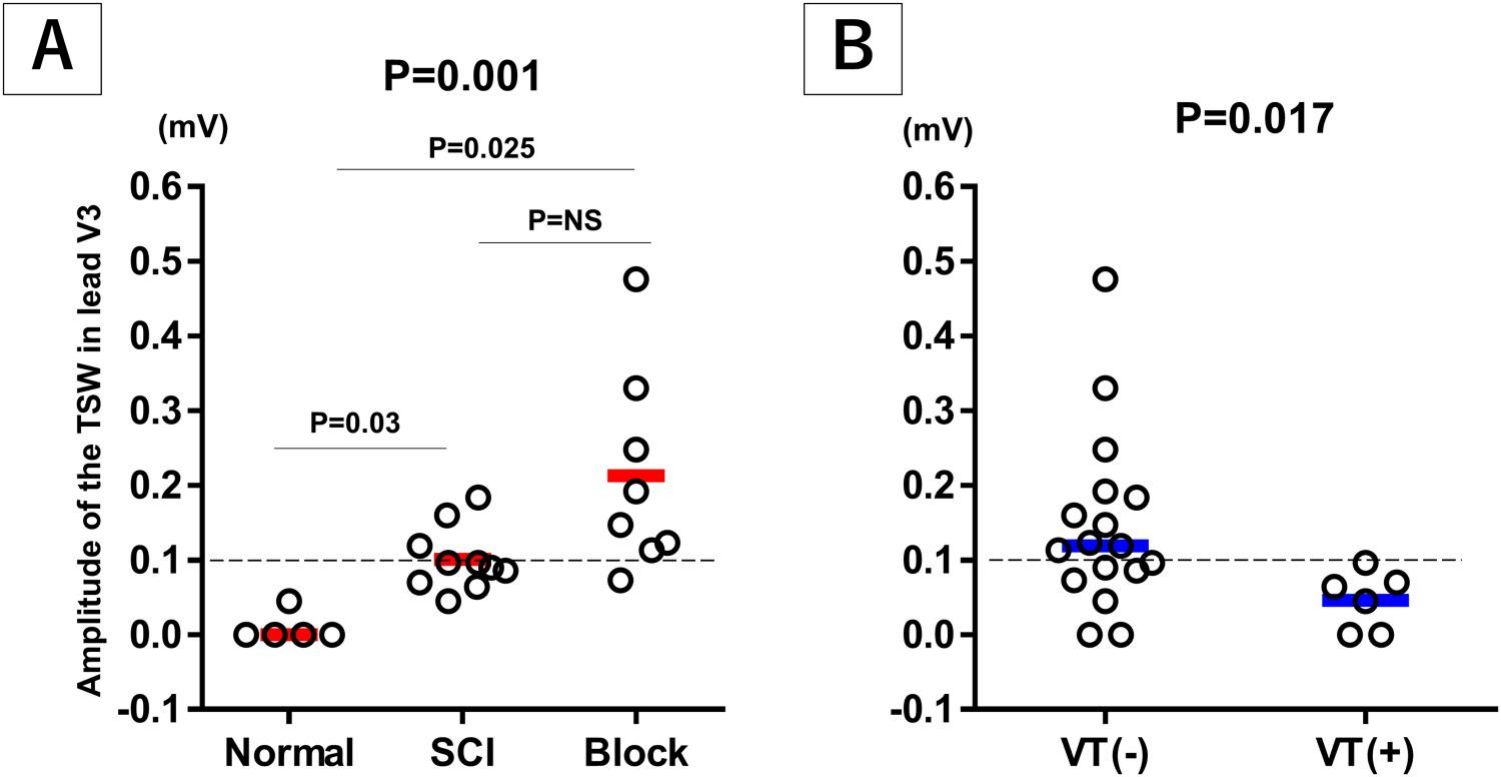
TSW amplitude in relation to conduction properties and VT inducibility. (A) Scatter plot of the terminal S wave (TSW) amplitude among groups classified based on the septal isthmus conduction property: normal conduction (Normal), slow‐conducting isthmus (SCI), and conduction block (Block). No statistically significant difference is observed between the SCI and Block groups, whereas the median value (red bar) is elevated in the Block group, with seven of eight patients exhibiting a TSW amplitude ≥ 0.1 mV. (B) Scatter plot of TSW amplitude in patients without inducible ventricular tachycardia (VT) and those with inducible VT. The median TSW amplitude was higher in the VT non‐inducible group than in the VT‐inducible group (*p* = 0.017). Notably, all patients with inducible VT had TSW values < 0.1 mV. SCI, slow‐conducting isthmus; TSW, terminal S wave; VT, ventricular tachycardia.

**Table 3:** Predictive value of TSW in V3 for the conduction property of the septal isthmus.

	Sensitivity	Specificity	PPV	NPV	*p*‐value
Small TSW (< 0.1 mV, excluding 0 mV) for predicting *SCI*	70%	85%	78%	79%	0.013
Large TSW (≥ 0.1 mV) for predicting *Block*	88%	80%	70%	92%	0.006
TSW < 0.2 mV, excluding 0 mV for predicting *SCI*	100%	54%	63%	100%	0.007
TSW ≥ 0.2 mV for predicting *Block*	38%	100%	100%	75%	0.032

Abbreviations: NPV, negative predictive value; PPV, positive predictive value; SCI, slow‐conducting isthmus; TSW, terminal S wave.

### 3.4 Predictive Value of TSW in Lead V3 for VT

VT was induced in 6/23 patients (26%) in the validation cohort.

The median TSW value was higher in the VT non‐inducible group than in the VT‐inducible group (0.12 vs. 0.05 mV, *p* = 0.017; Figure 2B). Although a small TSW (< 0.1 mV, excluding 0 mV) was not a significant predictor of VT inducibility (*p* = 0.162), all patients with inducible VT had TSW values below 0.1 mV (Figure 2B). Accordingly, ROC analysis for VT non‐inducibility yielded an AUC of 0.83 (*p* = 0.019), with an optimal cutoff of 0.073 mV, supporting 0.1 mV as a clinically practical threshold. Notably, a large TSW (≥ 0.1 mV) in lead V3 was a significant marker of VT non‐inducibility, with high specificity and a high positive predictive value (Table 4).

**Table 4:** Predictive value of TSW in V3 for VT inducibility.

	Sensitivity	Specificity	PPV	NPV	*p*‐value
Small TSW (< 0.1 mV, excluding 0 mV) for predicting VT inducibility	67%	71%	44%	86%	0.162
Large TSW (≥ 0.1 mV) for predicting *non‐inducibility of VT*	59%	100%	100%	46%	0.019

Abbreviations: NPV, negative predictive value; PPV, positive predictive value; TSW, terminal S wave; VT, ventricular tachycardia.

## 4 Discussion

In the present study, we demonstrated that ablation across the septal isthmus eliminated slow conduction and produced distinct ECG changes, most notably the accentuation of TSW in lead V3. The presence and amplitude of TSW in lead V3 were strongly associated with septal isthmus conduction properties, enabling noninvasive prediction of SCI or block. Furthermore, a large TSW was associated with VT non‐inducibility. Collectively, these findings suggest that the TSW in lead V3 may serve as a simple and practical ECG marker to facilitate risk stratification for VT in patients with rTOF.

Lead V3 overlies the RV free wall, and in rTOF, it approximately corresponds to the basal RV beneath the pulmonary artery (PA) (Figure 3, 1). In patients with CRBBB, delayed terminal activation toward the PA is reflected as a TSW in lead V3. When conduction block occurs across the septal isthmus, activation propagates from anterior to posterior, resulting in prominent delayed excitation toward the PA and a large TSW (Video 3, 1). In contrast, under normal conduction conditions, activation passes directly from the posterior septum to the PA, producing no delayed terminal deflection (Video 2). Slow conduction shifts the collision site more basally, attenuating the TSW (Figure 3, 1, Video 3). Notably, a small TSW predicted SCI, whereas a large TSW predicted conduction block. The substantial overlap between the SCI and Block groups and the limited sample size likely contributed to the absence of a statistically significant difference in TSW between the two groups. Nevertheless, seven of eight patients in the Block group in the validation cohort exhibited a TSW greater than 0.1 mV (Figure 2A). For all five patients included in the exploratory retrospective analysis study, the TSW increased after ablation and exceeded 0.1 mV. (Figure S4). These results indicate that a large TSW may be a useful marker of conduction block. Nonetheless, previously reported parameters, such as TSW in lead aVF or notched R in lead V1 [14, 15], were not clearly reproducible in our exploratory study, likely reflecting individual variability. Therefore, combining multiple ECG parameters may further enhance predictive accuracy.

**Figure 3:**
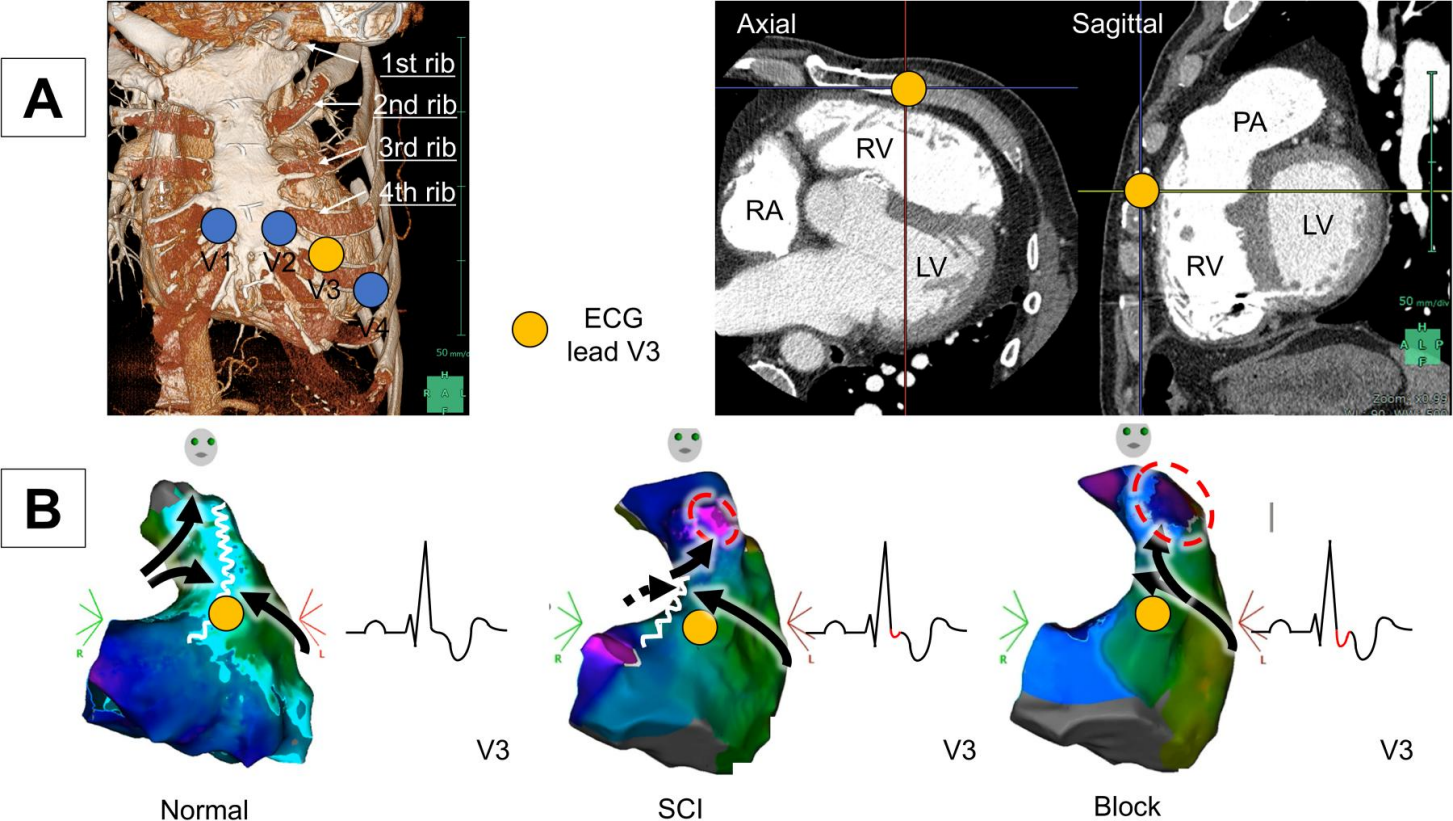
Relationship between lead V3 location and conduction properties of the septal isthmus. (A) Three‐dimensional CT reconstruction of chest anatomy depicting the ECG patch electrode positions on the body surface. The yellow circle indicates the location of lead V3. Lead V3 is located in the mid‐portion of the right ventricle, approximately beneath the pulmonary artery (PA). (B) Representative right ventricular (RV) activation maps. In normal conduction (*Normal*, left panel), the posterior and anterior wavefronts collide at the mid to basal RV free wall (white wavy line), corresponding to lead V3 (yellow tag). Activation from the posterior septum propagates directly toward the PA through the septal isthmus, and no delayed activation is observed within this region. In a slow‐conducting isthmus (*SCI*, middle panel), delayed and attenuated excitation toward the PA is observed (red dotted circle), likely accounting for the small TSW in lead V3. In the presence of a complete block line (*Block*, right panel), activation propagates from anterior to posterior, producing more direct excitation toward the PA and a large TSW in lead V3. (Videos [1–3]). LV, left ventricle; PA, pulmonary artery; RA, right atrium; RV, right ventricle; SCI, slow‐conducting isthmus; TSW, terminal S wave.

**Video 1:** Complete block of the septal isthmus and its relationship with the 12‐lead ECG. In the presence of a conduction block at the septal isthmus, activation of the right ventricular free wall propagated from anterior to posterior. Delayed excitation toward the pulmonary artery, propagating away from lead V3, resulted in a large terminal S wave (red dotted circle). PA, Pulmonary artery; TA, Tricuspid annulus.

**Video 2:** Normal conduction of the septal isthmus and its relationship with the 12‐lead ECG. In normal conduction, the posterior and anterior wavefronts collided at the mid‐to‐basal right ventricular free wall. Activation propagated directly toward the pulmonary artery through the septal isthmus without delayed activation, resulting in the absence of a terminal S wave in lead V3. PA, Pulmonary artery; TA, Tricuspid annulus.

**Video 3:** Slow conducting isthmus and its relationship with the 12‐lead ECG. In the presence of a slow‐conducting isthmus, delayed and attenuated excitation toward the pulmonary artery is observed (red dotted circle), corresponding to the appearance of a small terminal S wave in lead V3. PA, Pulmonary artery; TA, Tricuspid annulus.

SCI at the septal isthmus is a known cause of VT [8], and preventive ablation targeting this substrate can reduce the incidence of implantable cardioverter‐defibrillator therapies [16]. In the validation cohort, none of the patients with the block pattern had inducible VT. Furthermore, in all five exploratory patients who underwent ablation, a block line was successfully created across the septal isthmus, resulting in VT non‐inducibility. A large TSW in lead V3 was similarly associated with VT non‐inducibility. Collectively, these results suggest the potential value of TSW in lead V3 as a noninvasive marker for identifying septal isthmus block. This finding is clinically important because slow‐conducting septal isthmuses may serve as VT substrates, whereas blocked isthmuses are less likely to participate in reentrant VT circuits. Although a small TSW was associated with SCI in the septal isthmus in the present study, it did not serve as a significant marker of VT inducibility, possibly reflecting the limited sample size and variability in TSW. Accordingly, our findings should be considered hypothesis‐generating and should be further validated in larger, multicenter prospective cohorts.

This study has some limitations. First, the study population comprised patients under consideration for TPVI or surgical PVR, most of whom had moderate or greater pulmonary regurgitation and enlarged RVs. Consequently, they may not fully represent the broader rTOF population. Compared with previous reports, the prevalence of normally conducting anatomical isthmus 3 was lower in our cohort, whereas complete conduction block was more common [17]. This may reflect advanced postoperative remodeling with greater fibrosis and scar formation within the RVOT and anatomical isthmuses. Differences in surgical techniques and operative strategies may also have contributed; however, this could not be confirmed, as detailed surgical records were unavailable for many patients. In addition, RV enlargement may influence the relative positions of the precordial leads over the RV free wall; therefore, the lead showing the most prominent waveform changes may vary according to RV size and geometry. Furthermore, because the study was limited to patients with CRBBB, the findings may not be generalizable to patients with narrow QRS complexes.

Second, this was a single‐center study with a limited sample size. In particular, the number of patients with normally conducting septal isthmuses was small, which may have reduced the statistical power to distinguish these from slow conduction.

Third, detailed activation mapping or entrainment pacing during induced VT was not consistently performed in all patients in the validation cohort. Therefore, the relationship between induced VT and the septal anatomical isthmus could not be definitively confirmed in all cases.

Future analyses involving a larger cohort may further clarify the utility of V3 TSW as a predictor of VT inducibility or as a reassuring marker indicating a low likelihood of VT occurrence.

## 5 Conclusion

This study revealed that the pattern of TSW in lead V3 could reflect the conduction properties of the septal isthmus in patients with rTOF, suggesting its potential as a simple noninvasive marker for VT risk stratification.

## Disclosure

Dr. Igarashi received endowments from Japan Lifeline, Astec Co. Ltd., Medtronic, DVX, Boston Scientific, Abbott Medical Inc., and Biotronik. Dr. Komatsu received honoraria for lectures from Biosense Webster. The remaining authors have no conflicts of interest to disclose.

## Ethics Statement

This study complied with the Declaration of Helsinki and was approved by the University of Tsukuba Ethics Committee (R07‐027).

## Consent

The institutional review board waived the requirement for written informed consent, and an opt‐out method was used.

## Supporting information


Supporting File 1

Supporting File 2

Supporting File 3

Supporting File 4

Supporting File 5

## Data Availability

The data that support the findings of this study are available from the corresponding author upon reasonable request.
