# Supplementary material for: Terminal S Wave Pattern in Lead V3 as a Simple Marker for Ventricular Tachycardia Risk Stratification in Patients With Repaired Tetralogy of Fallot
Source: J Cardiovasc Electrophysiol. 2026 Aug 10;37(10):2261–9. doi: 10.1111/jce.70468 (PMC13661771; doi:10.1111/jce.70468)
Supplement: Supplementary file 2 [file jce70468-transc-0002-en.pdf]

Supplemental movie legends

Supplemental Movie S1. Complete block of the septal isthmus and its relationship with the 12-lead ECG.

In the presence of a conduction block at the septal isthmus, activation of the right ventricular free wall propagated from anterior to posterior. Delayed excitation toward the pulmonary artery, propagating away from lead V3, resulted in a large terminal S wave (red dotted circle).

PA, Pulmonary artery; TA, Tricuspid annulus

Supplemental Movie S2. Normal conduction of the septal isthmus and its relationship with the 12-lead ECG.

In normal conduction, the posterior and anterior wavefronts collided at the mid-to-basal right ventricular free wall. Activation propagated directly toward the pulmonary artery through the septal isthmus without delayed activation, resulting in the absence of a terminal S wave in lead V3. PA, Pulmonary artery; TA, Tricuspid annulus

Supplemental Movie S3. Slow conducting isthmus and its relationship with the 12-lead ECG.

In the presence of a slow-conducting isthmus, delayed and attenuated excitation toward the pulmonary artery is observed (red dotted circle), corresponding to the appearance of a small terminal S wave in lead V3. PA, Pulmonary artery; TA, Tricuspid annulus
