# Supplementary material for: Terminal S Wave Pattern in Lead V3 as a Simple Marker for Ventricular Tachycardia Risk Stratification in Patients With Repaired Tetralogy of Fallot
Source: J Cardiovasc Electrophysiol. 2026 Aug 10;37(10):2261–9. doi: 10.1111/jce.70468 (PMC13661771; doi:10.1111/jce.70468)
Supplement: Supplementary file 4 [file JCE-37-2261-s003.docx]

**Supplemental Table 1 Changes in ECG before and after ablation.**

| **ECG parameters** | | | **Before ablation** | **After ablation** |
| --- | --- | --- | --- | --- |
|  | S wave fragmentation in lead Ⅰ | | 3 (60) | 4 (80) |
|  | TSW in lead aVF | | 3 (60) | 5 (100) |
|  | R" in lead V1 | | 0 (0) | 0 (0) |
|  | Absence of TSW | in lead V2 | 4 (80) | 1 (20) |
|  | Small TSW |  | 0 (0) | 3 (60) |
|  | Large TSW |  | 1 (20) | 1 (20) |
|  | Absence of TSW | in lead V3 | **1 (20)** | 0 (0) |
|  | Small TSW |  | **3 (60)** | 0 (0) |
|  | Large TSW |  | **1 (20)** | **5 (100)** |
|  | Absence of TSW | in lead V4 | 0 (0) | 0 (0) |
|  | Small TSW |  | 1 (20) | 0 (0) |
|  | Large TSW |  | 4 (80) | 5 (100) |

Values are presented as numbers and n (%).

ECG, electrocardiogram; TSW, terminal S wave

**Supplemental Table 2**

|  | Case | anatomical isthmus 3 | anatomical isthmus 4 | **Septal isthmus** |
| --- | --- | --- | --- | --- |
| **exploratory study** | 1 | SCI | - | SCI |
|  | 2 | SCI | - | SCI |
|  | 3 | SCI | Block | SCI |
|  | 4 | SCI | - | SCI |
|  | 5 | SCI | - | SCI |
| **Validation cohort** | 1 | Normal | Normal | Normal |
|  | 2 | SCI | - | SCI |
|  | 3 | Normal | - | Normal |
|  | 4 | Block | - | Block |
|  | 5 | Block | Block | Block |
|  | 6 | Normal | - | Normal |
|  | 7 | Block | Block | Block |
|  | 8 | SCI | - | SCI |
|  | 9 | SCI | - | SCI |
|  | 10 | SCI | - | SCI |
|  | 11 | Block | - | Block |
|  | 12 | Block | - | Block |
|  | 13 | SCI | - | SCI |
|  | 14 | SCI | - | SCI |
|  | 15 | Normal | Normal | Normal |
|  | 16 | SCI | - | SCI |
|  | 17 | SCI | - | SCI |
|  | 18 | SCI | - | SCI |
|  | 19 | Normal | - | Normal |
|  | 20 | Block | - | Block |
|  | 21 | Block | - | Block |
|  | 22 | SCI | - | SCI |
|  | 23 | Block | - | Block |
